# Ocular inflammatory disease and ocular tuberculosis in a cohort of patients co-infected with HIV and multidrug-resistant tuberculosis in Mumbai, India: a cross-sectional study

**DOI:** 10.1186/1471-2334-13-225

**Published:** 2013-05-20

**Authors:** Salil Mehta, Homa Mansoor, Samsuddin Khan, Peter Saranchuk, Petros Isaakidis

**Affiliations:** 1 Ophthalmology Department, Lilavati Hospital and Research Centre, Mumbai, India; 2 Médecins Sans Frontières, Mumbai, India; 3 South African Medical Unit, Médecins Sans Frontières, Cape Town, South Africa

**Keywords:** HIV, TB-HIV, Multidrug-resistant tuberculosis, Ocular inflammatory disease, Ocular tuberculosis, Operational research, India

## Abstract

**Background:**

The prevalence and the patterns of ocular inflammatory disease and ocular tuberculosis (TB) are largely undocumented among Multidrug Resistant TB (MDR-TB) patients co-infected with Human Immunodeficiency Virus (HIV) and on antituberculosis and antiretroviral therapy (ART).

**Methods:**

Lilavati Hospital and Research Center and Médecins Sans Frontières (MSF) organized a cross-sectional ophthalmological evaluation of HIV/MDR-TB co-infected patients followed in an MSF-run HIV-clinic in Mumbai, India, which included measuring visual acuity, and slit lamp and dilated fundus examinations.

**Results:**

Between February and April 2012, 47 HIV/MDR-TB co-infected patients (including three patients with extensively drug-resistant TB) were evaluated. Sixty-four per cent were male, mean age was 39 years (standard deviation: 8.7) and their median (IQR) CD4 count at the time of evaluation was 264 cells/μL (158–361). Thirteen patients (27%) had detectable levels of HIV viremia (>20 copies/ml). Overall, examination of the anterior segments was normal in 45/47 patients (96%). A dilated fundus examination revealed active ocular inflammatory disease in seven eyes of seven patients (15.5%, 95% Confidence Intervals (CI); 5.1-25.8%). ‘These included five eyes of five patients (10%) with choroidal tubercles, one eye of one patient (2%) with presumed tubercular chorioretinitis and one eye of one patient (2%) with evidence of presumed active CMV retinitis. Presumed ocular tuberculosis was thus seen in a total of six patients (12.7%, 95% CI; 3.2-22.2%). Two patients who had completed anti-TB treatment had active ocular inflammatory disease, in the form of choroidal tubercles (two eyes of two patients). Inactive scars were seen in three eyes of three patients (6%). Patients with extrapulmonary TB and patients <39 years old were at significantly higher risk of having ocular TB [Risk Ratio: 13.65 (95% CI: 2.4-78.5) and 6.38 (95% CI: 1.05-38.8) respectively].

**Conclusions:**

Ocular inflammatory disease, mainly ocular tuberculosis, was common in a cohort of HIV/MDR-TB co-infected patients in Mumbai, India. Ophthalmological examination should be routinely considered in HIV patients diagnosed with or suspected to have MDR-TB, especially in those with extrapulmonary TB.

## Background

Ocular tuberculosis (OTB) results from the haematogenous dissemination of mycobacteria and may affect virtually any intraocular tissue. Clinical features depend on the specific tissue involved and may be due to both, direct tissue infection or due to hypersensitivity reactions. The characteristic findings include tubercles, tuberculomas and serpiginous-like choroiditis. These represent direct choroidal infection via the hematogenous route. Less common lesions include lupus vulgaris of the eyelids, conjunctivitis, corneal ulcers and phlyctenulosis, and scleritis. Neuro-opthalmological lesions include orbital apex syndrome, disc edema and sixth nerve palsies.

Non-invasive ocular examinations to detect ocular tuberculosis have several potential advantages. They may suggest a diagnosis in a subset of patients, thus allowing for a more focused investigational approach. This may be more important in immunocompromised patients who tend to have abnormal systemic findings (absence of fever) or investigational findings (anergic mantoux tests readings or unusual chest radiography). These findings may delay a diagnosis of systemic tuberculosis with the attendant risks of poor outcomes.

The association between systemic TB disease and ocular dissemination has been widely reported. The ocular morbidity pattern in 2010 eyes of 1005 patients with active pulmonary and extrapulmonary tuberculosis disease was studied prospectively in India; 1.4% of patients had ocular lesions [1]. In a study from Spain, Bouza et al. examined 100 patients with culture-positive tuberculosis and found that 18 (18%) of patients had tubercular choroiditis, papillitis, retinitis, and vitritis [2]. Eleven out of these 18 patients were HIV-infected. Systemic dissemination has also been reported to significantly increase the likelihood of ocular lesions up to 60% [3].

It is unclear whether the dual HIV-TB epidemic has lead to an increase in the prevalence and incidence of ocular tuberculosis in patients with active TB disease and co-infected with HIV. In various studies from Africa, differing proportions of ocular tuberculosis in association with HIV infection have been reported, varying between 0 to 2.8%, depending on the size and clinical characteristic of the studied cohort [4,5]. Opportunistic ocular infections are rare in an African population, despite the increasing availability of antiretroviral drugs with its attendant reduction of morbidity and mortality. In a prospective study from Mumbai, India, 23.5% of AIDS patients with systemic tuberculosis had ocular lesions [6] but a much lower prevalence was seen in a neighbouring city, where although as many as 66% of 1268 AIDS patients had systemic tuberculosis, only 1% of patients had ocular TB [7]. Babu et al., in the largest Indian cohort of referred HIV/AIDS patients (766) from a tertiary care centre, described ocular tuberculosis in 19 eyes of 15 patients (2.0%). Clinical presentations included choroidal granulomas in 10 eyes, subretinal abscess in seven eyes (worsening to panophthalmitis in three eyes), and conjunctival tuberculosis and panophthalmitis each in one eye [8].

Multidrug-resistant tuberculosis (MDR-TB) is defined as TB that is resistant to both isoniazid and rifampicin. Extensively drug-resistant tuberculosis (XDR-TB) is defined as MDR-TB with additional resistance to any fluoroquinolone and to at least one of three injectable second-line anti-tuberculosis drugs used in treatment (capreomycin, kanamycin or amikacin).

To-date, no data are available on the overall prevalence or common presentations of ocular inflammatory disease (OID), especially ocular tuberculosis, in patients with MDR-TB including those co-infected with HIV.

The objectives of this study were 1) to assess the prevalence and presentations of ocular inflammatory disease and ocular tuberculosis, in particular, in a cohort of MDR-TB patients co-infected with HIV and 2) to identify possible risk factors for developing ocular tuberculosis, including those factors related to HIV infection and to drug resistance.

## Method

### Ethics statement

The study was approved by the Institutional Review Board of Lilavati Hospital & Research Center, Mumbai, India and by the Médecins Sans Frontières independent Ethics Review Board, Geneva, Switzerland. Patients were counselled about the study and an informed consent was obtained.

### Study design

This was a cross-sectional study in a cohort of MDR-TB patients co-infected with HIV receiving care and treatment at an HIV clinic in Mumbai, India.

### Setting and study population

Médecins Sans Frontières (MSF) has been operating an HIV clinic in Mumbai since 2006 and has been treating MDR-TB among HIV-infected individuals since May 2007. MDR-TB treatment became available in the public sector in Mumbai only in late 2010. Patients received empirical or when possible individualized therapy through an ambulatory, community-based program that we have described elsewhere [9]. In summary, an individualized TB treatment regimen is designed for each patient, based on the first and second line drug susceptibility test (DST) results and on a patient’s treatment history. Patients are treated for at least 20 months, based on WHO guidelines updated in 2011 [10]. For treatment of HIV, first line antiretroviral therapy (ART) typically consists of two nucleoside/tide reverse transcriptase inhibitors (NRTIs) and one non-nucleoside reverse transcriptase inhibitor (NNRTI), while patients in need of second line ART received a protease inhibitor-based regimen along with NRTIs [11].

The study was conducted from February 2012 to April 2012. All MDR-TB patients co-infected with HIV who were followed in the MSF clinic at the time of the study were eligible to be included in the study, regardless if they were on treatment during the study period or had completed the treatment earlier. There were no exclusion criteria for this study; only patients who died, were lost to follow-up or were transferred out were not included in the study.

### Clinical evaluation protocol

Patients underwent a detailed clinical evaluation: patients were asked for systemic and ocular complaints, including self-reported ones that may have been due to antiretroviral and/or antituberculosis therapy. Systemic evaluation was conducted as per the HIV clinic follow-up protocol: this consisted of clinical assessment and laboratory monitoring, including complete and differential blood counts, baseline hepatic and renal function tests, HIV markers (subtype of infection, viral load and CD4 counts), sputum collection on two consecutive days or extrapulmonary biopsy (commonly lymph nodes) as necessary. All collected samples underwent smear studies (with Ziehl Neelsen stain), culture and drug susceptibility testing for first and second line anti-TB drugs when culture was positive. Appropriate radiography was also carried out.

Patients underwent a full ophthalmological evaluation by a consultant ophthalmologist familiar with ocular complications of HIV/AIDS, including visual acuity screening, slit lamp examination and dilated fundus examination of the entire retina in all patients using an indirect ophthalmoscope. The presence and severity of ocular inflammatory disease was assessed by documenting the presence, grade and type of anterior uveitis, presence and grade of vitreous cells (classified as grade 1, 2, 3 or 4), “snow-balls”, and the presence of cystoid macular oedema (CME), periphlebitis and intraocular pressure evaluation. We followed the currently accepted SUN (standardized uveitis nomenclature [12]) for the classification and grading of the ocular inflammation. Fundus fluorescein angiography or optical coherence tomography was done whenever deemed necessary by the physician.

### Data collection and analysis

Demographic and clinical information were systematically recorded in standardized clinical files. Information on HIV and antiretroviral therapy was entered into a specifically designed database. Data related to clinical evaluation of eyes were entered in a specific simple database. Data from all patients evaluated between February 2012 and April 2012 were included in the analyses. Patient characteristics were summarized using descriptive statistics. We used *t*-test, chi-square and Fisher’s exact test to assess differences between variables, as appropriate. Univariate and multivariate logistic regression and generalized linear models (GLM) were fitted to assess risk factors associated with the occurrence of ocular TB. Factors and covariates used as predictors included: age, sex, nadir CD4 count and CD4 count at the time of evaluation, HIV viral load, tuberculosis site (pulmonary/extrapulmonary), drug resistance pattern (MDR/XDR) and time on antiretroviral and anti-Tb treatment. We used the cohort’s mean age as a cut-off point in the modelling. For the parameter estimation in the generalized linear models we used the hybrid method and a robust estimator. The Wald statistic was used for the main analysis in the final model and the Wald type for the 95% confidence intervals. A p-value of less than 0.05 was considered to indicate statistical significance. The computer software SPSS (IBM, SPSS, Statistics, version 20) was used for analysis.

## Results

### Patient characteristics

A total of 51 patients were examined during the course of this study. No patient refused consent to participate. Four patients were excluded from the analyses; two HIV-infected patients were found to have pan-susceptible TB and two patients with MDR-TB were not HIV-infected, but were followed in the HIV clinic because they were household contacts of co-infected patients. Thus, forty-seven patients were included in the analyses: 28 (59.6%) males, 17(36.2%) females and two (4.2%) transgenders. Ages ranged from 12 years to 54 years (mean 39 years, SD: 8.7). The commonest subtype of HIV infection was type HIV-1 (44, 93.6%) followed by HIV-2 (2, 4.2%) and mixed HIV/1-2 infection (1, 2.1%). The median nadir CD4-count and the median CD4-count at the time of the evaluation were 153 cells/μL (IQR: 85–247) and 264 cells/μL (IQR: 158–361) respectively. Thirteen patients (27%) had detectable levels of HIV viremia (>20 copies/ml) at the time of the evaluation. Fourteen patients (24.8%) had been diagnosed with extrapulmonary TB (EPTB). The drug resistance patterns included 44 patients (93.6%) with multidrug resistance, and three patients (6.6%) with extensive drug resistance. Patients were on antiretroviral treatment for a median of 17.5 months (IQR: 4–32.5) and on second-line anti-TB treatment for a median of 13 months (IQR: 4–21) (Table 1).

**Table 1:** Patient characteristics (n=47)

**Characteristic**	
***Mean age (years) (SD)***	39 (8.7)
***Sex***	
Male, n (%)	30 (63.8)*
***HIV infection & Antiretroviral treatment***	
Median nadir CD4 count (cells/μL) (IQR)	153 (85–247)
Median CD4 count at the time of evaluation (cells/μL) (IQR)	264 (158–361)
HIV viral load undetectable (<20 copies/μL) at the time of evaluation, n (%)	34 (72.3)
Median duration of antiretroviral treatment (months) (IQR)	17.5 (4–32.5)
***Tuberculosis and anti-TB treatment***	
Multidrug-resistant TB (MDR-TB), n (%)	44 (93.6)
Extensively and extremely drug-resistant TB (XDR & XXDR-TB), n (%)	3 (6.4)
Extra-pulmonary TB	14 (29.8)
Median duration of anti-TB treatment (months) (IQR)	13 (4–21)

* Two biological males were transgender.

### Ocular inflammatory disease & ocular tuberculosis

Overall, examination of the anterior chamber was normal in 45/47 patients (96%). The examination of one patient (2%) showed a corneal opacity and one patient (2%) had a well functioning bleb from prior glaucoma surgery. Both patients were on ART and anti-TB treatment at the time of the study. Among 36 patients on ART and anti-TB treatment at the time of clinical evaluation and among 11 patients who had completed anti-TB treatment prior to the study, the examination of the anterior chambers was normal in 34 patients (94.4%) and in 11 patients (100%) respectively.

A dilated fundus examination revealed active or inactive ocular inflammatory disease in seven eyes of seven patients (15.5%, 95% Confidence Intervals (CI): 5.1-25.8%). These included five eyes of five patients (10.6%) with presumed choroidal tubercles (Additional file 1), three of whom were still on anti-TB treatment. Presumed tubercular chorioretinitis and CMV retinitis was each seen in one eye of one patient on treatment (2.1% respectively). Presumed ocular tuberculosis was thus seen in a total of six patients (12.7%, 95% CI: 3.2% to 22.2%). Of note, two patients who had completed anti-TB treatment had active ocular inflammatory disease, in the form of choroidal tubercles (two eyes of two patients).

Inactive scars were seen in three eyes of three patients (6.3%): one scar in one eye of a patient on treatment and two scars in two eyes of two patients who had completed treatment (one year and six months prior to this evaluation respectively). Other pathology seen included HIV retinopathy (two eyes of one patient, 2.1%) and secondary open angle glaucoma (one eye of one patient, 2.1%) not related to TB or HIV-infection. No cases of multifocal choroidopathy, vasculitis, anterior uveitis, intermediate uveitis or panuveitis were seen in the overall cohort. Table 2 summarizes the findings of the cross-sectional evaluation.

**Table 2:** Ophthalmological examination findings among HIV/MDR-TB co-infected patients in Mumbai, India (n=47)

**Findings**	**Eyes/patients (%)**
***Anterior segment***	
normal	90/45(96)
corneal opacity	1/1(2)
bleb	1/1(2)
***Posterior segment***	
normal	81/38(76)
tubercles	5/5(10)
scars	3/3(6)
chorioretinitis	1/1(2)
CMV retinitis	1/1(2)
HIV retinopathy	2/1(2)
Secondary open-angle glaucoma	1/1(2)

Visual screening showed acuities ranging from 6/6 to hand movements. Causes of visual loss included CMV retinitis, presumed tubercular chorioretinitis, corneal opacities and uncorrected refractive errors. All the patients with choroidal tubercles were visually asymptomatic whereas the patient with chorioretinitis had profound visual loss due to the macular location of the disease. No cases of elevated intraocular pressure were found. One patient (2.1%) was on topical antiglaucoma medication but the intraocular pressure was normal. We found no patients with ocular adverse events due to antiretroviral or antituberculosis drugs in this Mumbai cohort as previously reported [13].

### Risk factors for ocular tuberculosis

In univariate analyses, co-infected patients with extrapulmonary tuberculosis or patients with both pulmonary and extrapulmonary disease were eleven times more at risk of ocular TB compared with those having only pulmonary TB (risk ratio [RR] 11.62, 95% confidence interval [CI] 1.96–68.87, Table 3). Age below 39 years was associated with a five-fold increase in the prevalence of ocular TB, though this association was not statistically significant. Increased immunosuppression, as evidenced by the nadir CD4 T-cell count and CD4-count at the time of the evaluation, was not associated with heightened prevalence of ocular TB (Table 3). Gender, HIV viremia and time on anti-TB treatment did not show an association with ocular TB prevalence.

**Table 3:** Univariate and multivariate analysis of risk factors for ocular tuberculosis among HIV/MDR-TB co-infected patients (n=47)

**Factors**		**Risk ratio**	**95% Confidence intervals (CI)**	***p***	**Adjusted risk ratio**	**95% CI**	***p***
Sex	Male	1					
	Female	0.17	0.02- 1.51	0.11			
Age, years	>40	1			1		
	≤39	4.80	0.85-26.95	0.07	**6.38**	**1.05-38.80**	**0.04**
Nadir CD4-count	≤200	1					
	>200	0.65	0.13-3.21	0.60			
CD4-count at evaluation	≤200	1			1		
	>200	0.59	0.10-3.35	0.55	0.21	0.18-2.52	0.21
HIV viral load at evaluation	undetectable	1					
	detectable	1.18	0.20-6.75	0.85			
Tuberculosis site	pulmonary	1			1		
	extrapulmonary	**11.62**	**1.96-68.87**	**0.007**	**13.65**	**2.37-78.53**	**0.003**
Time on MDR-TB treatment (months)	>6	1			1		
	≤6	0.53	0.09-2.99	0.47	0.39	0.03-4.79	0.46

In multivariate analyses, extrapulmonary infection remained the most important determinant of ocular TB occurrence, i.e. with the greatest adjusted risk ratio (aRR: 13.65, 95% CI: 2.4-78.5, p=0.003). The only other factor independently associated with ocular TB was age below 39 years (aRR: 6.38, 95% CI: 1.05-38.8, p=0.04). The final multivariate model showed that immune status and duration of anti-TB treatment were not associated with the risk of ocular TB (Table 3).

## Discussion

In this study we report rates of ocular inflammatory disease and presumed ocular tuberculosis from a cohort of MDR-TB patients co-infected with HIV on antiretroviral and anti-TB treatment. Despite India’s large burden of MDR-TB and large absolute numbers of people living with HIV, there are discouragingly few reports of experiences with these patients [8,14,15]. To our knowledge this is the first report on ocular disease from co-infected patients in Asia and probably elsewhere.

We studied a relatively large cohort of HIV/MDR TB co-infected patients and found a fairly high prevalence of ocular inflammatory disease, commonest of which was presumed ocular tuberculosis followed by CMV retinitis. Other commonly described ocular inflammatory/infectious diseases in HIV infected patients such as toxoplasmosis or other viral retinitis (presumed herpes simplex/herpes zoster infections) were not seen in this study, probably due to effective antiretroviral therapy.

In this Mumbai cohort, we observed a high yield of ocular tuberculosis that is possibly due to the greater propensity of HIV/AIDS patients to develop tuberculosis, along with the hyperendemic disease patterns of tuberculosis seen in India. Patients who develop secondary MDR-TB (due to incorrect or inadequate regimens), as well those who contract primary MDR-TB, are likely to have a delay in diagnosis, with a corresponding extended period of mycobacteraemia, that increases the risk of ocular infection.

Our study reveals a higher prevalence of presumed ocular tuberculosis than that reported by Biswas et al. in a cohort of pulmonary tuberculosis patients in an era where drug resistance was presumably far less common, but less than that reported by Mehta et al. in a cohort of HIV/TB patients [1,6]. Both these reports and other similar studies did not address the issue of drug resistance in enrolled patients and this remains an unknown factor that does not permit accurate comparisons across these studies. It is possible that an increasing prevalence of drug-resistant tuberculosis, with or without HIV infection, is causing a corresponding increase in the prevalence of ocular tuberculosis.

The commonest pattern of ocular inflammation encountered in this Mumbai cohort was choroidal tubercles representing presumed choroidal tubercular infection, followed by presumed tubercular chorioretinitis. Posterior segment ocular tuberculosis in the form of multifocal choroidopathy or vasculitis was not seen in this cohort. Similarly, the classical patterns of granulomatous or non-granulomatous anterior, intermediate or panuveitis were not seen in any of the patients in our cohort. Our study differs significantly from Babu et al. who noticed a lower prevalence and a distinctly different clinical pattern. This is probably because we studied all patients irrespective of visual symptomatology, allowing us to detect asymptomatic patients having ocular tuberculosis without visual morbidity. The large institutional cohort reported by Babu and co-workers consisted of visually symptomatic referrals to a large tertiary care center, often after a significant delay.

Earlier reports from the developed world suggested mycobacterial ocular infections were much less common. In a study in 1987, tuberculosis was responsible for 0.2% of cases of posterior uveitis and no cases of anterior uveitis in Southern California [16]. Increasingly, more recent reports are identifying a resurgence of tuberculosis as the infecting organism in non-immunocompromised patients with ocular inflammatory disease. In Italy, recent studies have described the clinical and investigational features of cohorts of 35 and 53 patients [17,18]. In reports from Germany, PET/CT scan studies, being the imaging modality of choice, were able to suggest a tuberculous etiology in nine of 20 patients who tested positive in a quantiferon gold test. Mycobacterium tuberculosis was shown in cultures of lymph node biopsies in two patients. A good therapeutic response was achieved in nine of 11 (82%) patients with anti-TB therapy [19].

Among the risk factors we studied with univariate and multivariate models, age and extrapulmonary tuberculosis were independently and significantly associated with the occurrence of ocular TB. Widespread dissemination of mycobacterial infection, which is often seen in patients with extrapulmonary tuberculosis, may explain the higher risk for ocular TB possible risk factor. Interestingly, in this cohort of co-infected patients, severe immunosuppression, as evidenced in the nadir CD4 T-cell counts, was not associated with heightened prevalence of ocular TB.

Findings suggestive of ocular tuberculosis were recorded in two patients who had successfully completed their second line anti-TB regimen. These two patients underwent fluorescein angiography after an informed consent, and increased hyperfluorescence was seen suggestive of active intraocular inflammation. These patients, followed in our HIV clinic, are currently in good health, and are being closely monitored for the need for retreatment. Potentially, the use of ocular evaluation, in these groups of patients, may serve as a marker to monitor the therapeutic response. Larger cohorts that assess the ongoing ocular response of these patients may furnish data whether such patients may be better served by non-standard treatment regimens (therapeutic composition or duration) but such data are currently not available.

There are some limitations to this study. First, the study design was cross-sectional and the data were collected from a cohort in a specific programmatic setting. Our findings may not be applicable to other populations with HIV/MDR-TB co-infection. Second, even though this Mumbai cohort is relatively large, we suggest caution in the generalization of these study findings and the study of risk factors. However, considering the small size of the global MDR-TB cohort on treatment [12,13], our data yields important information regarding ocular inflammatory disease and ocular tuberculosis among HIV/MDR-TB patients in a resource-constrained setting. Third, the diagnosis of choroidal TB was presumptive, based on characteristic clinical findings. However, the presence of virtually pathognomonic findings (tubercles) in the context of systemic TB, as is the case in the Mumbai cohort, could be considered adequate for the diagnosis. The presence of other findings (vasculitis, retinitis etc.) is more contentious and may need specific microbiological or histopathological confirmation. Fourthly, the patients in our study were previously diagnosed with tuberculosis, thus the diagnostic utility of detecting ocular tuberculosis cannot be analyzed for this cohort.

The detection of ocular inflammatory disease in these patients has several potential applications. First, a protocol that mandates retinal screening of all those presenting with low CD4 counts, and at subsequent regular intervals in certain cases, may help in the early diagnosis of ocular infections (tuberculosis and other opportunistic infections) as well as assess for drug toxicity (e.g. linezolid-induced optic neuropathy). Since specialty ophthalmologic consultation is rarely available in resource-limited settings [20], we have investigated the possibility of HIV/AIDS clinicians performing indirect ophthalmoscopy at the primary care level as part of routine evaluation for OIs [21], and documented the feasibility of this strategy [22]. In support of this approach, all 6 cases of ocular TB in this report could have been detected in the HIV clinic by a trained HIV clinician performing dilated indirect ophthalmoscopy.

Second, assessment of tubercular healing may act as a clinical in vivo measure of systemic healing, even as an in vivo clinical tool for timing antitubercular therapy cessation, as compared to the current standard “one size fits all” duration of therapy. Investigating this possibility will require a prospective study correlating clinical outcomes with ocular outcomes documented with retinal photography.

Ophthalmologists will increasingly encounter drug-resistant forms of TB disease and need to be familiar with the disease patterns and second and third lines of anti-TB therapy. Clinicians providing care to MDR-TB patients co-infected with HIV and public health officials involved in programs for such patients should be aware of the ocular manifestations and the implications for them.

## Conclusions

In conclusion, based on our findings, we suggest that ophthalmological examination should be routinely considered in HIV patients diagnosed with or suspected to have MDR-TB, especially in those with extrapulmonary TB.

## Competing interests

The authors declare that they have no conflict of interest.

## Authors’ contributions

SM & PI conceived the study and wrote the first study protocol. PI & SM designed the study. SM collected the data with additional help from HM & SK. PI & SM analysed the data. SM & PI drafted the manuscript, and edited multiple versions with inputs from PS, HM & SK. All authors reviewed and approved the final version of the manuscript.

## Pre-publication history

The pre-publication history for this paper can be accessed here:

http://www.biomedcentral.com/1471-2334/13/225/prepub

## Supplementary Material

Additional file 1 A fundus photo of 40 year old male with HIV 1 and extrapulmonary and pulmonary multidrug resistant tuberculosis showing a choroidal tubercle close to the optic nerve head. Click here for file
