# Supplementary material for: Ocular inflammatory disease and ocular tuberculosis in a cohort of patients co-infected with HIV and multidrug-resistant tuberculosis in Mumbai, India: a cross-sectional study
Source: BMC Infect Dis. 2013 May 20;13:225. doi: 10.1186/1471-2334-13-225 (PMC3661345; doi:10.1186/1471-2334-13-225)

**Photo Legends**

**Photo 1.** A fundus photo of 40 year old male with HIV 1 and extrapulmonary and pulmonary multidrug resistant tuberculosis showing a choroidal tubercle close to the optic nerve head.


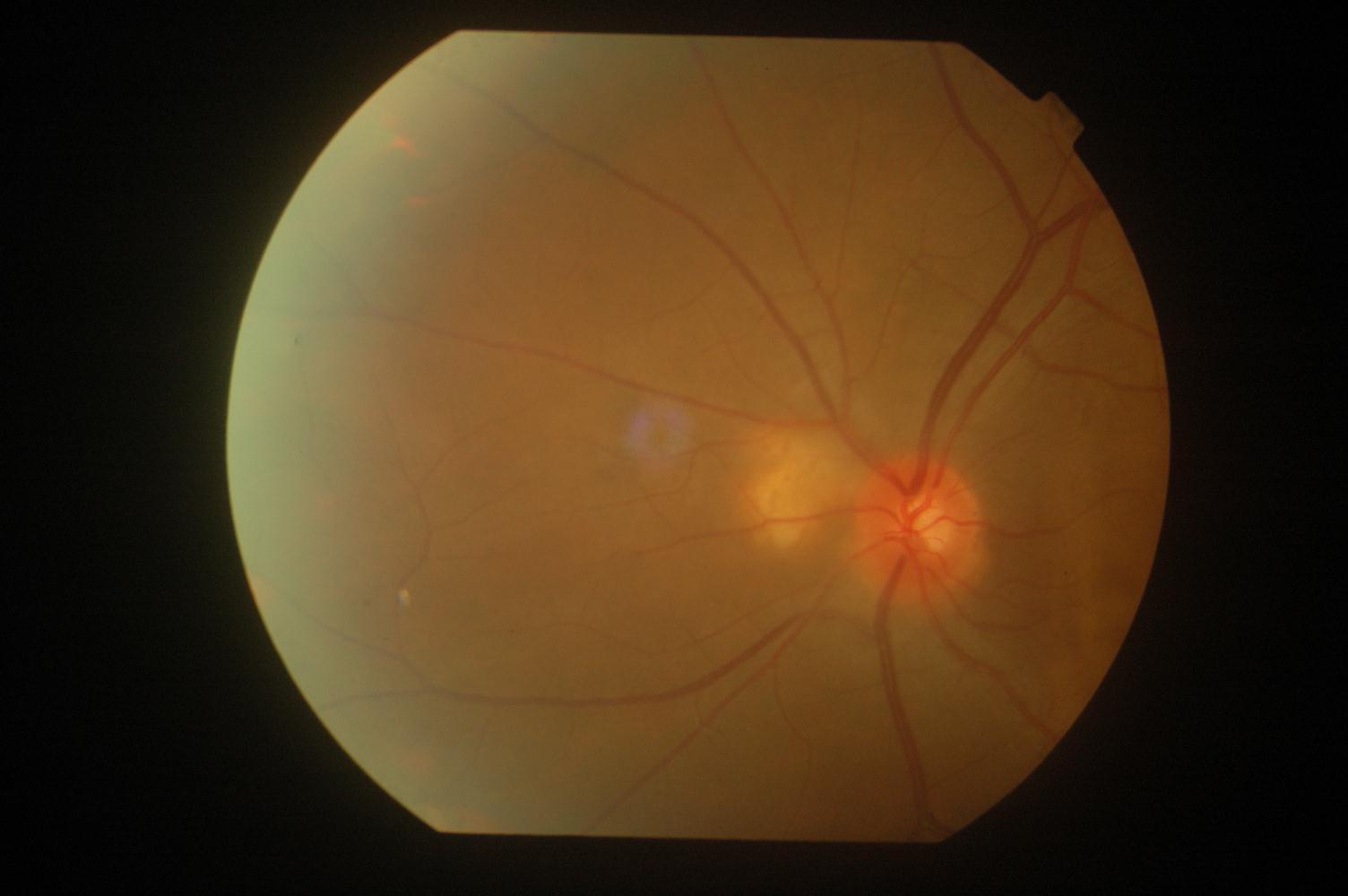

Supplement: Additional file 1 — A fundus photo of 40 year old male with HIV 1 and extrapulmonary and pulmonary multidrug resistant tuberculosis showing a choroidal tubercle close to the optic nerve head. [file 1471-2334-13-225-S1.doc]
